# Revisiting the Island of Doctor Moreau

**DOI:** 10.3201/eid2710.AC2710

**Published:** 2021-10

**Authors:** Byron Breedlove

**Affiliations:** Centers for Disease Control and Prevention, Atlanta, Georgia, USA

**Keywords:** art science connection, emerging infectious diseases, art and medicine, about the cover, the island of Doctor Moreau, revisiting the island of Doctor Moreau, Kate Gibb, H.G. Wells, War of the Worlds, viruses, bacteria, parasites, fungi, human encroachment, remote regions, factory farming, animal markets, trade, climate change, disruptions to natural areas, global migration, One Health, zoonoses

**Figure:**
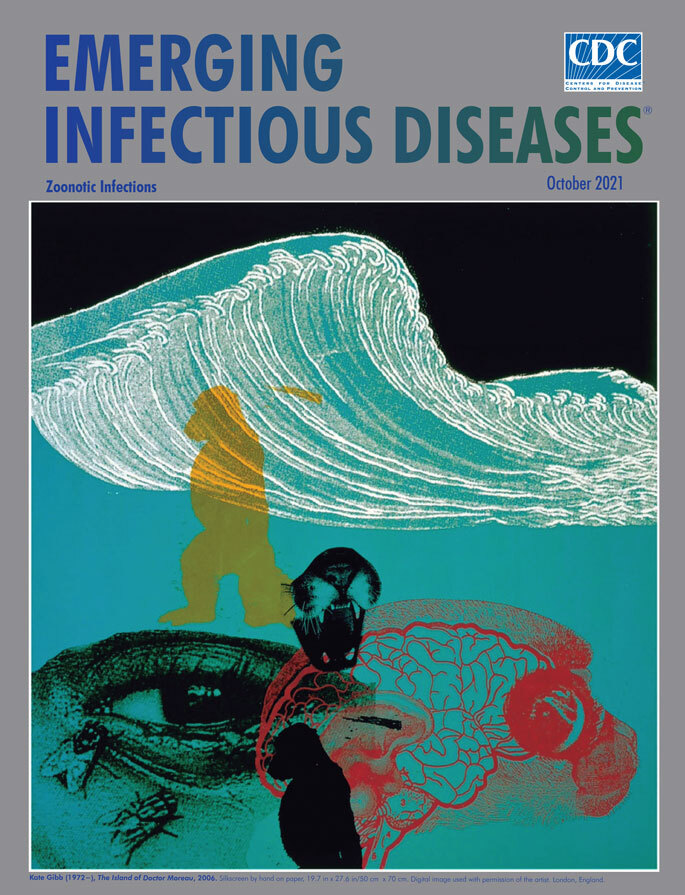
**Kate Gibb (1972–), *The Island of Doctor Moreau*, 2006.** Silkscreen by hand on paper, 19.7 in × 27.6 in/50 cm × 70 cm. Digital image used with permission of the artist. London, England.

This month’s cover art, created by contemporary English printmaker and illustrator Kate Gibb―known for her colorful, detailed screen-printed artwork developed for musicians and fashion designers―was first featured on another cover, the 2005 reissue of a classic work of early horror and science-fiction, *The Island of Doctor Moreau* by English writer H.G. Wells. Originally published in 1896, Wells’ novel chronicles the story of the shipwrecked Englishman Edward Prendick. Stranded on a remote, uncharted island, Prendick is nursed back to health after his ordeal at sea only to discover he has washed up on an isle of horrors. Doctor Moreau, a mad scientist, has fled to the island from England after his experiments in vivisection were exposed. Here the doctor works without restraints, accountability, or moral guidance, performing gruesome, cruel experiments designed to transform animals into humans.

In *The Island of Doctor Moreau,* Wells intertwines several societal themes from the latter part of the 19th century, including the growing antivivisection movement, implications of Charles Darwin’s research on evolution, the nature of humankind as explored in Robert Louis Stevenson’s 1886 novel *The Strange Case of Dr. Jekyll and Mr. Hyde*, and the notion of humans creating chimeras, an idea articulated earlier in Mary Shelley’s 1818 novel *Frankenstein*. Moreau’s horrific surgical procedures triggered ethical questions and concerns about cruelty, morals, and human–animal relationships that still resonate with contemporary readers. Literature professor Roger Luckhurst notes, “In the 21st century, with genetic splicing making animal–human hybrids an actual possibility, Wells’ queasy exploration of the limits of the human in this provocative satire keeps the book incredibly relevant today.” Moreau’s efforts to control the behaviors of his menagerie of engineered creations through “The Law,” a list of prohibitions recited to the island’s “Beast Folk” to keep them from reverting to their animalistic selves, are also troubling. 

Those who have read the novel will recall that after Moreau and his assistant Montgomery are killed, as are a number of the altered creatures, Prendick eventually escapes from the island and returns to London. He discovers, however, that he can no longer live among humans, fearing they, too, will revert to animalistic beings, and so he seeks solitude in the countryside, devoting himself to scientific studies. Gibb’s collage, which draws from the interconnected themes in the novel, features silhouettes of simians, the maw of a felid, and a lacey cross-section of a brain superimposed over a soothing blue-gray background, plus a pair of flies disturbingly close to a human eye peering at the viewer. 

As readers of this journal know, the xenotransplantation and surgeries, close interactions of humans and animals, and coerced association of myriad animal species on Moreau’s island could potentially have led to the spread of zoonotic infections, perhaps even the emergence of novel pathogens. But no person or animal is ever infected with a zoonotic pathogen in the novel, unlike in Wells’ next novel, *The War of the Worlds*, when invading Martians succumb to earthly pathogens to which they had no immunity, “slain, after all man’s devices had failed. . . .”

Zoonotic diseases, an unavoidable consequence of human–animal interactions, are caused by microorganisms such as viruses, bacteria, parasites, and fungi. CDC’s One Health website notes that more than 6 out of every 10 known infectious diseases in humans can be spread from animals. Perhaps in a contemporary reimaging of *The Island of Doctor Moreau*, existing or emerging zoonotic infections would factor into the narrative. In such a reworking, the isolated island setting and small number of people and animals might mitigate widespread transmission of such infections, in contrast with factors driving the emergence of new infectious diseases. Human encroachment into remote regions, factory farming, animal markets and trade, climate change, disruptions of natural areas and their ecosystems, and global migration all play a role. 

Understanding those interconnections between humans, animals, plants, and their communal environment, a concept now known as One Health, is an important public health priority. In a 2018 article published in the *Annual Review of Animal Biosciences*, researchers Bird and Mazet warn, “We must be prepared to recognize the signs, identify the threat, and rapidly work together to reduce the spread of infections and health consequences before they harm the health of animals and people throughout the world.” Gibb’s silkscreened image seems applicable both to the events in Well’s 125-year-old novel and to those of the present day.

## About the Artist

Kate Gibb created 17 covers for the 2006 reissues of H.G. Wells’ works and explained, “There were strong themes and descriptive elements within the text which quickly inspired me to find and collage images quite spontaneously. I remember really enjoying the process and being pleasantly surprised by its outcome!” She has worked with fashion designers, publishers, and musicians, and her work has been featured in a number of contemporary publications. In addition to producing her print-based artworks and commercial illustrations, Gibb is an educator, most recently at England’s University of Brighton. After studying textiles at Middlesex University, London, she shifted her focus to silkscreen printing and describes herself as “a silkscreen obsessive.”

## References

[1] Bird BH, Mazet JAK. Detection of emerging zoonotic pathogens: an integrated One Health approach. Annu Rev Anim Biosci. 2018;6:121–39. 10.1146/annurev-animal-030117-014628 29144769

[2] Bishop A. Making sympathy “vicious” on *The Island of Dr. Moreau.* Ninet Century Contexts. 2021;43:205–20. 10.1080/08905495.2021.1898229

[3] Breedlove B, Arguin PM. Anthropomorphism to zoonoses: two inevitable consequences of human–animal relationships. Emerg Infect Dis. 2015;21:2282–3. 10.3201/eid2112.AC2112

[4] Centers for Diseases Control and Prevention. One Health [cited 2021 Aug 17]. https://www.cdc.gov/onehealth/index.html

[5] Gibb K. About [cited 2021 Aug 17]. http://kategibb.co.uk

[6] Gibb R, Redding DW, Chin KQ, Donnelly CA, Blackburn TM, Newbold T, et al. Zoonotic host diversity increases in human-dominated ecosystems. Nature. 2020;584:398–402. 10.1038/s41586-020-2562-8 32759999

[7] Jones KE, Patel NG, Levy MA, Storeygard A, Balk D, Gittleman JL, et al. Global trends in emerging infectious diseases. Nature. 2008;451:990–3. 10.1038/nature06536 18288193 PMC5960580

[8] Luckhurst R. An introduction to the island of Dr. Moreau: science, sensation and degeneration [cited 2021 Aug 17]. https://www.bl.uk/romantics-and-victorians/articles/an-introduction-to-the-island-of-doctor-moreau-science-sensation-and-degeneration

[9] Wells HG. The island of Doctor Moreau. [cited 2021 Aug 7] https://www.gutenberg.org/files/159/159-h/159-h.htm

[10] +81 Gallery. Kate Gibb: October 19–November 19, 2017 [cited 2021 Aug 25]. http://www.plus81.us/exhibition/kate-gibb

